# Deep learning techniques for imaging diagnosis of renal cell carcinoma: current and emerging trends

**DOI:** 10.3389/fonc.2023.1152622

**Published:** 2023-09-01

**Authors:** Zijie Wang, Xiaofei Zhang, Xinning Wang, Jianfei Li, Yuhao Zhang, Tianwei Zhang, Shang Xu, Wei Jiao, Haitao Niu

**Affiliations:** ^1^ Department of Vascular Intervention, ShengLi Oilfield Center Hospital, Dongying, China; ^2^ Department of Education and Training, The Affiliated Hospital of Qingdao University, Qingdao, China; ^3^ Department of Urology, Affiliated Hospital of Qingdao University, Qingdao, China; ^4^ Extenics Specialized Committee, Chinese Association of Artificial Intelligence (ESCCAAI), Beijing, China

**Keywords:** deep learning, artificial intelligence, carcinoma, prediction model, imaging diagnosis

## Abstract

This study summarizes the latest achievements, challenges, and future research directions in deep learning technologies for the diagnosis of renal cell carcinoma (RCC). This is the first review of deep learning in RCC applications. This review aims to show that deep learning technologies hold great promise in the field of RCC diagnosis, and we look forward to more research results to meet us for the mutual benefit of renal cell carcinoma patients. Medical imaging plays an important role in the early detection of renal cell carcinoma (RCC), as well as in the monitoring and evaluation of RCC during treatment. The most commonly used technologies such as contrast enhanced computed tomography (CECT), ultrasound and magnetic resonance imaging (MRI) are now digitalized, allowing deep learning to be applied to them. Deep learning is one of the fastest growing fields in the direction of medical imaging, with rapidly emerging applications that have changed the traditional medical treatment paradigm. With the help of deep learning-based medical imaging tools, clinicians can diagnose and evaluate renal tumors more accurately and quickly. This paper describes the application of deep learning-based imaging techniques in RCC assessment and provides a comprehensive review.

## 1 Introduction

Renal cell carcinoma (RCC) is one of the most common and fatal tumors of the urinary system. It originates from the urinary tubular epithelial system of the renal parenchyma and accounts for 4% of human malignancies. Its annual incidence exceeds 400,000 cases, with a total of approximately 431,288 cases worldwide in 2020 (1). Clear cell RCC (ccRCC) is the predominant type of RCC pathology. RCC is usually detected on computed tomography (CT) scans, and it is estimated that about 15-40% of patients are found incidentally while undergoing CT examinations (2, 3). RCC is usually asymptomatic in its early stages, and approximately 25-30% of patients present with metastases at the time of diagnosis. Early diagnosis of RCC will significantly improve prognosis; therefore, with the increasing number of RCC cases, it is critical to develop effective strategies for early diagnosis and identification of tumors with poor prognosis (4).

Deep learning is a branch of machine learning techniques. Traditional machine learning techniques include support vector machine (SVM), random forest, decision tree, K-nearest neighbor, naive Bayes, logistic regression, etc. (5, 6). The emergence of convolutional neural networks (CNN) has raised the accuracy of machine learning to a new level. As models continue to iterate in complexity, machine recognition capabilities are reaching human levels for the first time (7) which has led to the explosion of deep learning applications today. Deep learning technologies are starting to change various fields of production and life, such as AlphaGo, Face Payment, and Autopilot, which are well known to the public.

**Figure 1:**
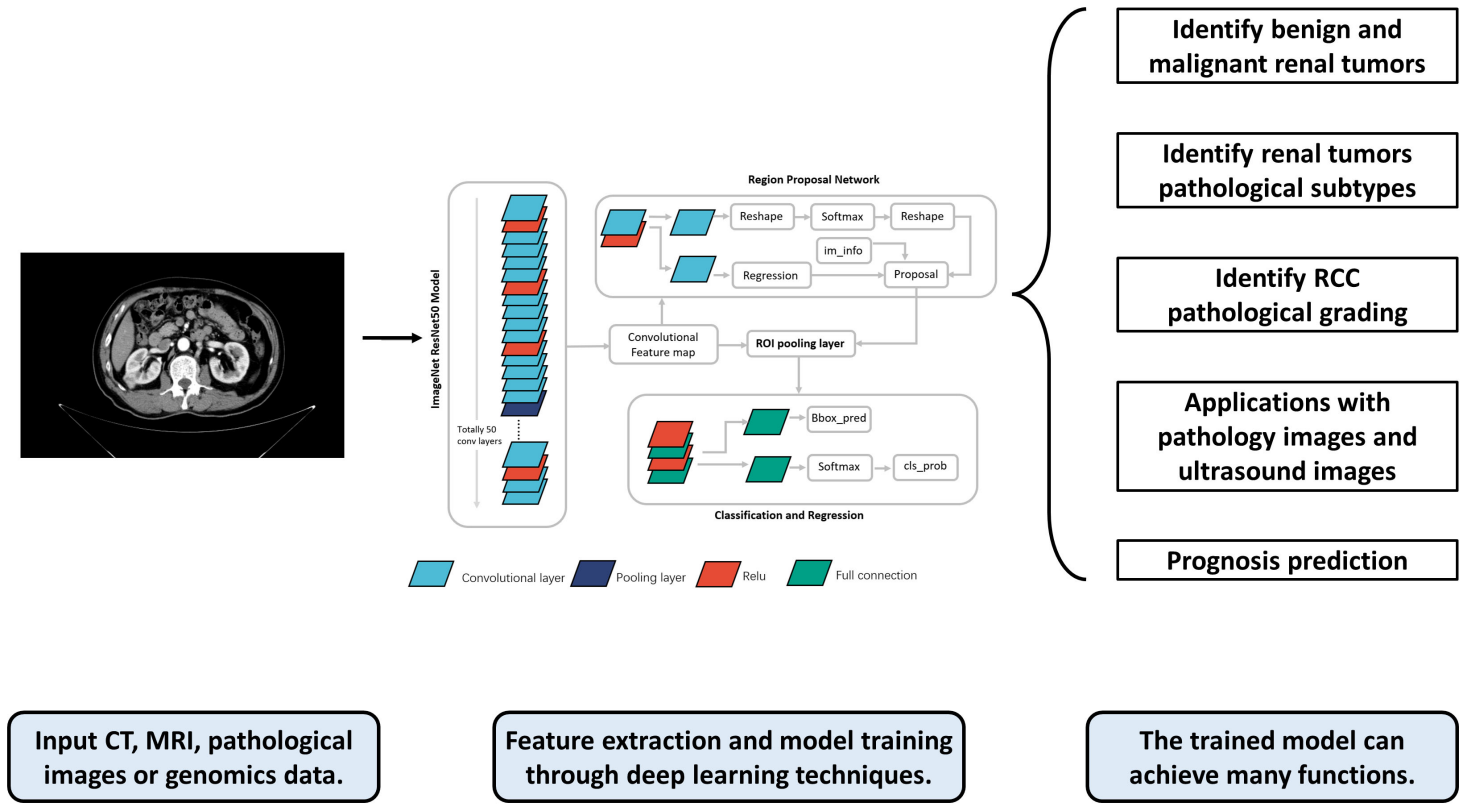
The flowchart and application overview of deep learning research. First, data such as radiological, pathological, and genomic data from patients are collected as inputs. These data are preprocessed and fed into the deep learning model for training. The trained model can output a variety of prediction results, such as the pathological grade, pathological type, and prognosis of RCC patients, providing references for doctors’ subsequent diagnosis and treatment.

With the rapid development of computer hardware and deep learning theory, deep learning has been widely used for the classification of medical image processing (8). Currently, deep learning models have achieved diagnostic accuracy for most tumor images at the level of radiologists. (e.g., rectal cancer (9), breast cancer (10), lung cancer (11), etc.). CNNs and improved models have been widely used for medical image processing (12). In the field of urology, deep learning-based predictive models have achieved excellent results in the diagnosis and treatment of various diseases such as RCC, prostate cancer (13–15), bladder cancer (16–18), and urolithiasis (19–21). This paper summarizes the research on deep learning in the areas of pathological identification, pathological grading, and prognostic treatment of RCC, and discusses its future research directions. The flowchart and application overview of deep learning research can be seen in **Figure 1**.

## 2 Deep learning to identify benign and malignant renal tumors

It is important to have an accurate imaging description of renal tumors because not all incidental findings of renal tumors are RCC. Up to 20% of solid renal tumors less than 4 cm in size are benign, most commonly renal oncocytoma (RO) and renal fat-poor angiomyolipoma (fpAML) (22). Currently, methods to differentiate between benign and malignant renal tumors are still limited. Although a percutaneous biopsy can confirm the diagnosis in most cases, it is relatively invasive. Studies have shown (23) that there is a risk of biopsy channel implantation in renal tumors (1.2%), especially in papillary RCC (pRCC) (12.5%). Therefore, as stated in the EAU (24), the “small but real” risk of channel implantation must be weighed in patients with renal tumors when puncture is necessary to determine subsequent treatment options. Also, although relatively uncommon, complications of renal tumor biopsy (e.g., hematoma, back pain, severe hematuria, pneumothorax, and hemorrhage) should not be ignored as well (25). Therefore, an ideal method for the diagnosis of renal tumors should ensure a high accuracy and detection rate while avoiding unnecessary potential risks to patients as much as possible. This calls for further improvements in complementary diagnostic techniques to increase sensitivity and specificity. The preoperative image diagnosis system constructed based on deep learning is mostly trained with pathological results as the golden standard, and the accuracy can often reach more than 90%. Its application in clinical practice can help patients avoid the risk of puncture and improve the diagnosis rate significantly (See **Table 1**).

**Table 1:** Summary of studies on the identification of renal tumor subtypes.

Author	Publication Date	Research Objectives	Imaging Type	Patients	DL model	Predicted Outcome Accuracy
Zhou, L (26).	2019	benign & malignant tumor differentiation	CECT: CM or CP or EX	134 malignant (117ccRCC, 8 pRCC, 9 other) & 58benign (50 renal cysts, 8 AML)	Inception-v3	The model had an accuracy of 97%, a sensitivity of 95% and a specificity of 97%
Tanaka, Takashi (27)	2020	benign & malignant tumor differentiation (tumor ≤ 4 cm)	four‐phasic CECT	32 benign (11 RO, 20 fpAML, 1 other) & 136 malignant (117 ccRCC, 7 pRCC, 6 chRCC, 4 other)	Inception-v3	The CM phase images achieved highest accuracy (88%)
Zabihollahy, F (28).	2020	benign & malignant tumor differentiation	CECT: UN CM CP	77 benign (57 RO & 20 fpAML) and 238 malignant (123 ccRCC & 69 pRCC & 46 chRCC)	CNN made up of 6 Conv. layers	The semi-automated majority voting-based CNN algorithm achieved accuracy, precision, and recall of 83.75%, 89.05%, and 91.73%
Xi, I. L (29).	2020	benign & malignant tumor differentiation	MRI: T2WI T1C	655 malignant & 507 benign	ResNet-50	Ensemble deep learning model had high test accuracy (0.70), sensitivity (0.92), and specificity (0.41).
Han, S (30).	2019	distinguish three major subtypes (ccRCC, pRCC, chRCC)	CECT: UN CM CP	57 ccRCC & 56 pRCC & 56 chRCC	GoogLeNet	The network showed about 0.85 accuracy, 0.64-0.98 sensitivity, 0.83-0.93 specificity, and 0.9 AUC.
Zheng, Y (31).	2021	renal tumor subtype differentiation	MRI: T2WI	77 ccRCC & 42 pRCC & 46 chRCC & 34 AML	ResNet	The model had a 60.4% overall accuracy, a 61.7% average accuracy, and a macro-average AUC of 0.82. The AUCs for ccRCC, chRCC, AML, and pRCC were 0.94, 0.78, 0.80, and 0.76, respectively.
Zuo, T (32).	2021	classification of pRCC & chRCC	CECT: CM CP EX	42 PRCC & 38 ChRCC	MobileNetV2, EfficientNet, ShuffleNet, ResNet-34, ResNet-50, ResNet-101	The best model achieved 96.8640% accuracy (99.3794% sensitivity and 94.0271% specificity) in the validation set and 100% (case accuracy) and 93.3333% (image accuracy) in the test set. The manual classification achieved 85% accuracy (100% sensitivity and 70% specificity) in the test set.
Lee, H (33).	2018	classification of fpAML & ccRCC	CECT	39 fpAML & 41 ccRCC	AlexNet,VGGNet, GoogleNet, ResNet	AlexNet model achieved the highist accuracy of 76.6%
Oberai, A (34).	2020	classification of fpAML & RCC	four‐phasic CECT	46 fpAML & 97 RCC	a simple CNN architecture	The CNN-based classifier demonstrated an overall accuracy of 78% sensitivity of 70%, specificity of 81% and an AUC of 0.82.
Coy, H (35).	2019	classification of RO & ccRCC	four‐phasic CECT	128 ccRCC & 51 RO	Google TensorFlow™ Inception	Classification performance was best in the EX-phase with an accuracy of 74.4%, a sensitivity of 85.8% and a PPV of 80.1%
Baghdadi, A (36).	2020	classification of RO & chRCC	CECT: UN CM CP	212 renal masses	NiftyNet	Model achieved accuracy of 95% in tumour type classification (100% sensitivity and 89% specificity)
Pedersen, M (37).	2020	classification of RO & RCC	CECT: UN CM CP	369 patients	ResNet-50 V2	Test_1 AUC of 0.973 with 93.3% accuracy and 93.5% specificity. Test_2 AUC of 0.946 with 90.0% accuracy and 98.0% specificity.
Nikpanah, M (38).	2021	classification of RO & ccRCC	MRI: T2WI T1WI	203 ccRCC & 40 RO	AlexNet	Overall accuracy of the AI system was 91% with an AUC of 0.9.

RCC, renal cell carcinoma; ccRCC, clear cell RCC; pRCC, papillary RCC; chRCC, chromophobe RCC; RO, renal oncocytoma; fpAML, fat-poor angiomyolipoma; DL, deep learning; CNN, convolutional neural networks; CECT, contrast enhanced computed tomography; UN, unenhanced; CM, corticomedullary; NP, nephrographic; EX, excretory; AUC, area under the curve; MRI, magnetic resonance imaging.

Zabihollahy, F. et al. (28) included 77 benign renal tumors (57 RO, 20 fpAML) and 238 malignant renal tumors (123 ccRCC, 69 pRCC, 46 chromophobe RCC (chRCC)) to construct a model, which was based on a self-created semi-automatic and fully automatic method (39) to segment the tumor from normal renal tissue. Ultimately, the semi-automatic method achieved 83.75%, 89.05% and 91.73% accuracy, precision and recall on the test set, respectively. The fully automated method obtained 77.36%, 85.92%, and 87.22% accuracy, precision, and recall, respectively.

Tanaka, Takashi et al. (27) wanted to identify benign and malignant at the scale of small renal tumors ≤ 4 cm, they collected four-phase contrast enhanced CT (CECT) data of 168 renal tumors and trained 6 models (unenhanced (UN), corticomedullary (CM), nephrographic (NP), and excretory (EX) phase, enhanced three-phases, and all four-phases), respectively, using the Inception-v3 architecture CNN model, And finally the highest accuracy (88%) was found for the NP phase images, with an area under the subject operating curve (ROC) (AUC) of 0.846.

Magnetic resonance imaging (MRI) is suitable for patients allergic to intravenous CT contrast agents and pregnant women and has a better function than CECT for the assessment of inferior vena cava involvement. Xi, I. L. et al. (29) included data from 1162 renal lesions to develop a deep learning model by applying a residual network (ResNet) on MRI (T1C and T2WI) to distinguish benign renal tumors from RCC. The accuracy (0.70), sensitivity (0.92), and specificity (0.41) of the deep learning model were significantly higher than those of the radiomics model as well as the expert models.

## 3 Deep learning to identify RCC pathological subtypes

According to the type of pathology, 60% to 80% of RCC are ccRCC and the rest are non-ccRCC. The World Health Organization (WHO) has developed a total of 4 versions of renal tumor classification criteria, and the current one is followed by the introduction of the fourth version of tumor classification criteria in 2016 (40). The growth pattern, treatment options, and risk of recurrence vary among different pathological subtypes of tumors. For example, AML, RO, renal cyst, cystic renal cancer, and other lesions can be followed up and observed. Precise preoperative evaluation of such tumor pathology can reduce unnecessary surgical treatment. Targeted therapy and immunotherapy also need to change the type and dose of drugs according to the pathological subtype of the tumor. In conclusion, if the pathological type of renal tumor can be known preoperatively, patients can benefit significantly.

Coy, H. et al. (35) used the open source Google TensorFlow™ Inception model to discriminate between RO and ccRCC, and three-phase CECT data as well as coronal, sagittal, and horizontal data were incorporated into the training model, achieving a positive predictive value of 82.5%. Biopsy differentiation between RO and chRCC currently remains a challenge, as both have similar molecular characteristics in addition to the typical histological features of tumor cells. Baghdadi, A. et al. (36) constructed an original predictive metric that can discriminate RO from chRCC on CECT images by measuring the tumour-to-cortex peak early-phase enhancement ratio (PEER) (41). They automatically identified tumor types by building deep learning algorithms to automatically measure the metric. The authors also introduced the concept of Dice similarity score (DSS) to quantitatively evaluate the difference between the model outline and the expert outline as another indicator of the model accuracy. The PEER assessment achieved 95% accuracy (100% sensitivity and 89% specificity) in the classification of tumor types compared to actual pathology results.

PRCC and chRCC are the most common types of non-ccRCC. Differences in origin factors and driver genes between the two have led to different treatment options and prognosis (42). PRCC and chRCC have some differences in imaging findings. PRCC presents with cysts, necrosis, and calcification, whereas chRCC presents with central whorl-like enhancement (43). However, in early stage or small sized masses, these aforementioned features are atypical and usually cause diagnostic difficulties. Teng et al. (32) used a total of six deep learning models to identify pRCC and chRCC. They extracted four case samples from The Cancer Imaging Archive (TCIA), a public database of cancer images, to participate in forming an external test set, and the best model (MobileNetV2) achieved 96.9% accuracy in the validation set (99.4% of sensitivity and 94.1% of specificity) and 100% (case accuracy)/93.3% (image accuracy) in the test set. Han, S. et al. (30) constructed a multiclassification model to discriminate ccRCC, pRCC, and chRCC based on the GoogLeNet model, the network showed an accuracy of 0.85, sensitivity of 0.64-0.98, specificity of 0.83-0.93, and AUC of 0.9.

## 4 Deep learning to identify RCC pathological grading

The Fuhrman grading system is highly recognized in the field of oncology diagnosis and is widely used in the pathological grading of ccRCC (44). In 2012, the International Society of Urological Pathology (ISUP) introduced a new grading system for ccRCC and pRCC (45), which was incorporated into the latest World Health Organization (WHO) classification of renal tumors and designated as the WHO/ISUP grading system (40). In this grading system, tumors are classified into four different grades (I, II, III and IV), with higher grades indicating more severe disease. The automatic classification of pathology using deep learning methods can significantly reduce the workload of pathologists, and the acquisition of pathology grading based on preoperative imaging data can help urologists to develop fine treatment strategies earlier, significantly improving patient survival and reducing suffering (See **Table 2**).

**Table 2:** Summary of studies predicting the pathological grading of ccRCC.

Author	Publication Date	Research Objectives	Imaging Type	Patients	DL model	Predicted Outcome Accuracy
Zhao, Y (46).	2020	Differentiating low-grade (grade I-II) from high-grade (grade III-IV) in stage I and II	MRI: T2WI T1C	376 patients with 430 RCC lesions	ResNet-50	Model achieved a test accuracy of 0.88, sensitivity of 0.89, and specificity of 0.88 in the Fuhrman test set and a test accuracy of 0.83, sensitivity of 0.92, and specificity of 0.78 in the WHO/ISUP test set.
Xu, L (47).	2022	Differentiating low-grade (grade I-II) from high-grade (grade III-IV)	CECT: UN CM CP	706 ccRCC	RegNet-400, RegNet-800, ResNet-50, ResNet-101	Single model AUC of 0.864, ensembled model AUC of 0.882.
Lin, F (48).	2020	Differentiating low-grade (grade I-II) from high-grade (grade III-IV)	CECT: UN CM CP	410 ccRCC	ResNet-18, ResNet-34, ResNet-50	In the external test, the DL model achieved an ACC and AUC of 77.9% and 0.81, respectively.
Yang, M (49).	2022	Differentiating low-grade (grade I-II) from high-grade (grade III-IV)	CECT: UN CM CP	759 ccRCC	TransResNet	The integrated model acquires a better performance (86.5% ACC and an AUC of 0.912).

RCC, renal cell carcinoma; ccRCC, clear cell RCC; DL, deep learning; CNN, convolutional neural networks; CECT, contrast enhanced computed tomography; UN, unenhanced; CM, corticomedullary; NP, nephrographic; EX, excretory; AUC, area under the curve; MRI, magnetic resonance imaging.

Lin, F. et al. (48) classified WHO/ISUP classification I and II as low grade and III and IV as high grade. They then trained ResNet models based on CECT images and achieved good results on both internal validation set (accuracy=73.7, AUC=0.82) and external test set (accuracy=77.9, AUC=0.81).

Xu, L. et al. (47) first validated the model on data from a large cohort, where they used a cohort containing 706 ccRCC patients to construct a deep learning model to predict Fuhrman classification. The traditional model was also refined by adding a two-step process of mixed loss strategy and sample reweighting to identify high-grade patients with ccRCC, to dealing with the domain shift problem and the noisy label problem, as well as the imbalance dataset problem. They developed 4 deep learning networks separately and further combined different weights for better prediction. In the validation cohort, the AUC of the single deep learning model is 0.864, while the AUC of the integrated model is 0.882.

Zhao, Y. et al. (46) evaluated the efficacy of ResNet using MRI in discriminating between high and low grade RCCs in a sample of patients with AJCC grade I and II. 353 Fuhrman-graded RCCs were divided into training, validation, and test sets in a ratio of 7:2:1. 77 WHO/ISUP-graded RCCs were used as separate test sets. Finally, the Fuhrman test set achieved 0.88 accuracy, 0.89 sensitivity, and 0.88 specificity, the WHO/ISUP test set achieved 0.83 accuracy, 0.92 sensitivity, and 0.78 specificity.

## 5 Deep learning combined with traditional radiomics

Radiomics, derived from texture analysis technology, is a technique for diagnostic prediction by extracting features from image data with high throughput and filtering them to build models, usually using traditional machine learning methods to model the filtered features.

With the advent of deep learning techniques, some studies have used self-constructed or mature CNNs to model the extracted radiomics features (33, 50). There are many differences between traditional machine learning-based radiomics and deep learning-based radiomics. Traditional radiomics relies on manually designed feature extraction and traditional machine learning algorithms to analyze medical image data. These features may include shape, texture, intensity, and so on. Traditional machine learning algorithms such as Support Vector Machines (SVM) and Random Forest are used to train models, which are then applied to tasks such as classification, segmentation, prediction, etc. Deep learning-based radiomics, on the other hand, utilizes neural network structures for automatic feature learning and pattern recognition. Deep learning models can learn high-level abstract features through multiple layers of neural networks, eliminating the need for manual feature extraction. This ability for automatic learning allows deep learning-based radiomics to perform well in handling large-scale and complex medical image data. Furthermore, the performance of traditional machine learning methods is often limited by the quality and selection of features, whereas deep learning-based radiomics can directly learn the optimal feature representation from raw data through an end-to-end training and optimization process, resulting in better performance.

## 6 Deep learning in pathology images, ultrasound images and other fields

Identifying histological differences in different RCCs under the microscope is a time-consuming and labor-intensive task for pathologists. There is also a high rate of variation of inter- and intra-observer by manual identification of RCCs (51) Kidney tumors can have different appearance and combination morphologies, making them difficult to classify. With the advent of whole section images in digital pathology, automated histopathology image analysis systems have shown great promise for diagnosis (52–54). Computerized image analysis has the advantage of providing a more valid, objective, and consistent assessment to assist pathologists in their diagnosis. Deep learning-based models that automatically process digitized histopathology images and learn to extract cellular patterns associated with the presence of tumors can assist pathologists by (1) automatically pre-screening sections to reduce false-negative cases, (2) highlighting important areas on digitized sections to expedite diagnosis, and (3) providing objective and accurate diagnoses (See **Table 3**).

**Table 3:** Summary of other applications of deep learning in renal tumor.

Author	Publication Date	Research Objectives	Imaging Type	Patients	DL model	Predicted Outcome Accuracy
Tabibu, S (55).	2019	renal tumor pathology tissue differentiation	pathological slide images	1027 ccRCC, 303 pRCC, and 254 chRCC, 477 normal tissues	ResNet-18, ResNet-34	Models distinguish ccRCC and chRCC from normal tissue with a classification accuracy of 93.39% and 87.34%, respectively. Model trained to distinguish ccRCC, chRCC and pRCC achieves a classification accuracy of 94.07%.
Zhu, M (56)	2021	renal tumor pathology tissue differentiation	pathological slide images	456 malignant slide images (cRCC, pRCC, chRCC), 30 normal images slides (RO, and normal)	ResNet-18, ResNet-34, ResNet-50, ResNet-101	The average AUC of our classifier on the internal resection slides, internal biopsy slides, and external TCGA slides is 0.98, 0.98 and 0.97, respectively.
Abu Haeyeh, Y (57).	2022	renal tumor pathology tissue differentiation	pathological slide images	25 ccRCC, 15 ccpRCC, 7 renal parenchyma, and 5 fat tissues	ResNet-50	Model achieves an overall classification accuracy of 93.0%, a sensitivity of 91.3%, and a specificity of 95.6%, in distinguishing ccRCC from ccpRCC or non-RCC tissues.
Zhu, D (58).	2022	benign & malignant tumor differentiation	CEUS images	81 benign and 100 malignant	MUF-Net	MUF-Net achieved accuracy of 80.0%, sensitivity of 80.4%, specificity of 79.1%, and AUC of 0.877, respectively.
Schulz, S (59).	2021	prognosis prediction	pathological slide images, CT/MRI scans, and genomic data from whole-exome sequencing	248ccRCC	ResNet	Model achieves an average C-index of 0.7791 and an average accuracy of 83.43%

RCC, renal cell carcinoma; ccRCC, clear cell RCC; pRCC, papillary RCC; chRCC, chromophobe RCC; ccpRCC, clear cell papillary RCC; RO, renal oncocytoma; DL, deep learning; CNN, convolutional neural networks; CEUS, contrast-enhanced ultrasound.

Zhu, M et al. (56) developed a deep learning model that accurately classifies digitized surgical and biopsy sections into five relevant categories: ccRCC, pRCC, chRCC, RO, and normal tissue. Their test set included 78 surgical resection full sections, 79 biopsy sections from the same institution, and 917 surgical resection sections from The Cancer Genome Atlas (TCGA) database. The mean AUC of the model on internal surgical sections, internal biopsy sections, and external TCGA sections was 0.98, 0.98, and 0.97, respectively. Abu Haeyeh, Y. et al. (57) trained three multi-scale CNNs and applied decision fusion to their predictions to obtain the final classification decision. For four types of kidney tissues: non-RCC renal parenchyma, non-RCC adipose tissue, ccRCC and clear cell papillary RCC (ccpRCC). The developed system showed high classification accuracy and sensitivity at the slide level for RCC biopsy samples, with an overall classification accuracy of 93.0%, sensitivity of 91.3%, and specificity of 95.6%.

A recent systematic review and meta-analysis (60) compared the diagnostic performance of enhanced ultrasound (CEUS) with CECT in the assessment of benign and malignant renal masses. 16 studies were included in the pooled analysis and the results showed comparable diagnostic performance with CEUS versus CECT (sensitivity 0.90 vs. 0.96). There are relatively few deep learning discrimination systems based on RCC ultrasound images, but several studies have been applied to assess the severity of hydronephrosis (61–63), It shows that deep learning techniques also have strong diagnostic efficacy for ultrasound images of the kidney. Zhu, D et al. (58) developed a deep learning model for CEUS images, called multimodal ultrasound fusion network (MUF-Net), and a total of 9794 images were cropped from CEUS videos for automatic classification of benign and malignant solid renal tumors. The performance of the model was compared with different experience levels radiologists. Accuracy was 70.6%, 75.7%, and 80.0% for the junior radiologist group, senior radiologist group, and MUF-Net, respectively, with AUC of 0.740, 0.794, and 0.877, respectively.

## 7 Deep learning in prognosis prediction

Utilizing deep learning techniques for predicting the prognosis of renal cancer can provide clinical doctors with more accurate patient risk assessment and treatment decision support, avoiding over-treatment or delayed treatment. Furthermore, the automated feature learning and prediction capabilities of deep learning models have the potential to enhance the efficiency and speed of prognosis assessment, offering practical solutions for large-scale prognosis evaluation of renal cancer patients.

Currently, there are limited studies on deep learning-based prognosis prediction for renal tumors. Schulz, S et al. (59) were the first to train a model on multi-scale data, incorporating histopathological images, CT/MRI scans, and genomic data from whole-exome sequencing of 248 patients. They developed and evaluated a multimodal deep learning model (MMDLM) for predicting the prognosis of clear cell renal cell carcinoma (ccRCC). The model achieved promising results, with an average C-index of 0.7791 and an average accuracy of 83.43%. However, the study also has certain limitations, such as missing imaging data for some patients and a relatively small dataset.

## 8 Discussion

In recent years, deep learning techniques have made significant progress in a wide range of computer vision tasks as well as biomedical imaging analysis applications. Deep learning techniques have been integrated into the medical industry for several years and have shown significant value in the diagnosis, identification, and staging of RCC, but there are still many areas of research that have yet to be broken through by deep learning techniques. The following are some possible future research directions.

### 8.1 Research for predicting patient prognosis

Prognostic analysis of tumor patients is an important application of deep learning research, but the current deep learning research in the field of RCC mostly stays at the level of diagnosis and identification. There is limited research on predicting the prognosis of RCC patients. Studies on the efficacy of immunotherapy and targeted therapy for RCC patients are still lacking.

### 8.2 Combined with molecular biology data

Radiomics combined with genomics has formed radiogenomics, where the presence of high expression of specific genes in patients can be discerned by identifying their preoperative images, such as PET/MRI-based identification of VEGF genes (64), CT-based identification of PBRM1, BAP1, and VHL gene mutation levels (65–68), and also combined proteomics studies (69). Such studies not only extend the boundaries of deep learning prediction models, but also add a plausible biological explanation of deep learning at the molecular level to deepen our understanding of how deep learning works. Subsequent studies could update the machine learning models in the above studies to deep learning models to significantly improve prediction accuracy.

### 8.3 Evaluate other imaging indicators of RCC

Deep learning technology combined with clinical diagnosis and treatment still has many areas in urgent need, especially the evaluation of some clinicopathological fine indicators. Similarly in the field of rectal cancer, in addition to the traditional benign-malignant differentiation and TNM staging rating, indicators such as circumferential resection margin(CRM) status (70) and tumor budding (71) have also become hot spots, and their role in guiding patient prognosis remains indispensable. In the field of RCC radiomics research, there are similar studies that have not yet been transplanted to deep learning models, such as Juxtatumoral perinephric fat invasion (72), inferior vena cava tumor thrombosis and vessel wall invasion (73), and evaluation of perirenal fat adhesions (74). Methodologically, these studies are no longer difficult to perform, only that no studies have been published yet.

### 8.4 Combined with cutting-edge imaging technology

An emerging area in RCC imaging is the use of pharmacokinetics from dynamic contrast-enhanced MRI. By dynamically tracking the distribution and clearance of MRI contrast agents, pharmacokinetic analysis can provide important information about tumor blood flow, vascular permeability, and extracellular space, which is extremely valuable for the diagnosis and differential diagnosis of renal cell carcinoma. For instance, the study by Wang et al. (75) has demonstrated the potential of pharmacokinetic parameters in differentiating subtypes of RCC and determining the malignancy of tumors. Deep learning techniques, especially CNN have been applied to analyze DCE-MRI data, to automatically extract and learn these pharmacokinetic parameters, thereby further improving the diagnostic accuracy of renal cell carcinoma. However, this field still faces some challenges, such as how to accurately extract pharmacokinetic parameters from various dynamic sequences, and how to address the issue of time and spatial resolution in dynamic enhancement data. Future research needs to address these issues and further explore the application of deep learning in the pharmacokinetic analysis of DCE-MRI in RCC.

Deep learning techniques have a wide range of promising applications in various clinical disciplines, but many challenges remain before the relevant results can be translated into clinical applications.

### 8.5 Mostly single-center studies

Most of the studies conducted so far are from the same medical center and have not been fully validated in independent cohorts, which leads to biased results and reduces the generalizability of the studies. We still need more multicenter, randomized controlled trials to enhance testing. Multidisciplinary and extensive cooperation to actively promote the maturation, standardization, and clinical development of deep learning research.

### 8.6 Insufficient number of patients

As a field combined with medical big data, enough data is a prerequisite for establishing models and a guarantee for maintaining stable system performance. Current studies in hotspot areas are mostly around 100-200 cases, there are still some risks of overfitting, and it is urgent to establish a platform for sharing large data of multi-center images.

### 8.7 Lack of prospective studies

The current studies in various hot areas are mostly retrospective, lacking large samples of randomized multicenter prospective tests, and there is still a large gap with the actual clinical application.

### 8.8 Lack of unified standard

The process of deep learning image acquisition lacks a unified standard or evaluation system, and the comparability of various studies of the same type is poor due to many reasons such as imaging equipment parameters, image construction, imaging physician habits and patient compliance.

### 8.9 Lack of repeatability

Image segmentation is an essential step in the deep learning model building process, and the repeatability of manual, semi-automatic, and automatic methods vary and has its own advantages and disadvantages, so how to improve the outlining accuracy with high repeatability is the current problem to be optimized. Both overfitting and underfitting of data can affect the repeatability of the model and optimization of algorithm is still the breakthrough of innovation in this field.

### 8.10 Higher requirements for multidisciplinary communication

Since the training of deep learning models requires high-throughput data processing, traditional statistical methods and analysis tools used in clinical research are no longer competent, which puts higher demands on the interdisciplinary ability and communication level of radiologists, surgeons, and computer engineers. There is still a need to figure out how doctors can better interface with engineers.

### 8.11 Self-supervised learning techniques have received limited research attention

Self-supervised learning can partially address the issue of data scarcity, especially in segmentation tasks. In traditional supervised learning, a large amount of labeled data is required for model training, which is costly and time-consuming to obtain. In contrast, self-supervised learning techniques leverage unlabeled data by designing tasks that generate labels automatically or utilizing unsupervised tasks. This allows models to learn meaningful features and semantic information from the unlabeled data. The advantage of self-supervised learning lies in its ability to enhance model performance, reduce reliance on many labeled data, and accelerate the training process by fully leveraging unlabeled data. It provides a valuable solution for coping with data scarcity.

### 8.12 Difficulties in deep learning model explainability

Deep learning models have achieved impressive results in the medical field, but their explainability remains a challenge. Deep learning models typically consist of multiple layers of neural networks, with many parameters and complex nonlinear mapping relationships. This complexity leads to opaque decision-making processes, making it difficult to explain the basis for their predictions. This lack of explainability can raise issues of trust and acceptance in medical practice. To address this problem, researchers have proposed various strategies, and one important approach is using Grad-CAM (Gradient-weighted Class Activation Mapping) (76). Grad-CAM is a gradient-based interpretability method that associates the model’s prediction results with specific local regions in the input image. Grad-CAM determines which regions in the image are crucial for a specific prediction result by computing the gradient of the predicted class with respect to the last convolutional layer. It then visualizes these key regions on the image to help doctors or researchers understand the basis of the model’s decisions. Such visualizations provide an intuitive display of the areas the model pays attention to during the prediction process, offering some explanatory power for the model’s decisions. In addition to Grad-CAM, there are other methods and techniques used to enhance the interpretability of deep learning models, such as LIME (Local Interpretable Model-agnostic Explanations), SHAP (SHapley Additive exPlanations) (77), and more. These methods attempt to analyze the model’s prediction results from different perspectives, providing explanatory insights and increasing the trustworthiness and acceptability of the model in medical practice.

## 9 Conclusion

In this paper, we conducted a comprehensive review of the latest advancements and challenges in the use of deep learning techniques for the imaging diagnosis of renal cell carcinoma. Through the analysis of various deep learning models in the application of renal cell carcinoma imaging diagnosis, we found that these technologies have enormous potential, significantly improving the accuracy and efficiency of diagnosis. However, these methods also have some limitations, such as the availability and quality of data, the interpretability of the models, and challenges in clinical applications. Despite these challenges, we believe that with the further development and improvement of deep learning techniques, their applications in the imaging diagnosis of renal cell carcinoma will become increasingly widespread. We look forward to more research in the future to overcome existing challenges and further promote the development of this field.

## Author contributions

ZW, JL, and WJ: Project development. TZ, XZ, and YZ: literature review and data extraction. ZW, TZ and XW: manuscript drafting. WJ, and HN: critical revision of the manuscript. All authors contributed to the article and approved the submitted version.
